# Low-level laser therapy prevents medication-related osteonecrosis of the jaw-like lesions via IL-1RA-mediated primary gingival wound healing

**DOI:** 10.1186/s12903-022-02678-1

**Published:** 2023-01-10

**Authors:** Yi Zheng, Xian Dong, Shuo Chen, Yang He, Jingang An, Meng Liu, Linhai He, Yi Zhang

**Affiliations:** 1 Department of Oral and Maxillofacial Surgery, Peking University School and Hospital of Stomatology, 22 Zhongguancun Nandajie, Haidian District, Beijing, 100081 People’s Republic of China; 2 First Clinical Division, Peking University School and Hospital of Stomatology, 22 Zhongguancun Nandajie, Haidian District, Beijing, 100081 People’s Republic of China; 3 Laser and Cosmetic Surgery Division, Peking University School and Hospital of Stomatology, Beijing, People’s Republic of China

**Keywords:** Medication-related osteonecrosis of the jaw, Zoledronate, Low-level light therapy, Interleukin-1 receptor antagonist, Interleukin-1 beta, Tumor necrosis factor-α, Primary gingival wound healing

## Abstract

**Background:**

Medication-related osteonecrosis of the jaw (MRONJ) is a serious debilitating disease caused by anti-resorption and anti-angiogenesis drugs, significantly affecting patients' quality of life. Recent studies suggested that primary gingival wound healing may effectively prevent the development of MRONJ. This study aimed to evaluate the effects of low-level light therapy (LLLT) on promoting gingival wound healing in extraction sockets of MRONJ-like mice and preventing the occurrence of MRONJ. Furthermore, we explored underlying mechanisms.

**Methods:**

Mice were randomly divided into the Ctrl, Zol, and Zol + LLLT groups. Administration of zoledronate and tooth extraction of bilateral maxillary second molars were used to build the MRONJ model, and LLLT was locally administered into the tooth sockets to examine the effect of LLLT. Next, to explore the function of IL-1RA, we performed LLLT with interleukin-1 receptor antagonist (IL-1RA) neutralizing antibody (named Zol + LLLT + IL-1RA NAb group) or negative control antibodies for tooth extraction in subsequent rescue animal experiments. Stereoscope observations, micro-computed tomography, and histological examination were conducted to evaluate gingival wound healing and bone regeneration in tooth sockets. The effects of LLLT on the migration capacities of zoledronate-treated epithelial cells were assessed in vitro.

**Results:**

LLLT promoted primary gingival wound healing without exposed necrotic bone. Micro-computed tomography results showed higher bone volume and mineral density of the tooth sockets after LLLT. Histology analysis showed complete gingival coverage, obvious bone regeneration, and reduced soft tissue inflammation, with down-regulated pro-inflammation cytokines, like interleukin-1 beta (IL-1β) and tumor necrosis factor-α (TNF-α), and up-regulated IL-1RA expression in the gingival tissue in the LLLT group. The rescue assay further showed that the effects of LLLT promoting gingival wound healing and preventing MRONJ might be partially abolished by IL-1RA neutralizing antibodies. In vitro studies demonstrated that LLLT accelerated zoledronate-treated epithelial cell migration.

**Conclusions:**

LLLT might promote primary gingival wound healing and contribute to subsequent bone regeneration of the tooth extractions in MRONJ-like lesions via IL-1RA-mediated pro-inflammation signaling suppression.

**Supplementary Information:**

The online version contains supplementary material available at 10.1186/s12903-022-02678-1.

## Introduction

Medication-related osteonecrosis of the jaw (MRONJ) is a severe adverse drug reaction associated with antiresorptive or antiangiogenic medication used to manage osteoporosis, lung, breast cancers, multiple myeloma, and cancer-related bone metastasis [1]. Patients are considered to harbor MRONJ if the following characteristics are present: (1) exposed bone in the maxillofacial region persisted for > 8 weeks; (2) current or previous treatment with antiresorptive or antiangiogenic agents; (3) no history of radiation therapy to the jaws or obvious metastatic disease to the jaws [1]. This condition is usually challenging to treat and can cause substantial pain and reduce the quality of life. Also, the pathogenic mechanisms of MRONJ remain elusive. Currently, the proposed pathophysiology hypostasis of MRONJ includes bone remodeling inhibition, infection, angiogenesis inhibition, immune dysfunction, gene factors, and so on [1, 2]. Most reported cases of MRONJ were caused by dental operations, like tooth extractions, intraoral surgical intervention, and mucosal trauma [3, 4].

Recent studies have suggested bisphosphonates (BPs)-induced impaired soft tissue healing as a potential mechanism of MRONJ. In 2017, Hasegawa et al*.* recommended the removal of any bone edges and mucosal wound closure as standard procedures in patients receiving bisphosphonates [5]. In 2019, we applied adipose-derived stem cells to prevent the onset of bisphosphonate-related osteonecrosis of the jaw in rabbits through transforming growth factor-beta-1 (TGF-β1)-mediated gingival wound healing [6]. It has been reported that surgery in the early stages can ensure favorable outcomes in terms of mucosal integrity and lesion downstaging [7]. Moreover, a few other studies suggested promoting primary soft tissue healing or closure after surgical operation to effectively reduce the occurrence of MRONJ [8–10]. Therefore, well-healed soft tissue could be essential for preventing the onset of MRONJ.

Previous studies have found that photobiomodulation (PBM) and low-level laser therapy (LLLT) can regulate critical cellular pathways and energetic cellular metabolism mediated by ATP, calcium, or reactive oxygen species [11]. In addition, numerous studies have indicated that LLLT may be a potentially appealing biophysical non-invasive method that contributes to wound healing by establishing homeostasis, reducing pain and inflammation, and boosting collagen accumulation, wound granulation, and revascularization [12, 13]. LLLT has been applied for treating various disease conditions like diabetic wounds [14, 15], bone repair [16], neuronal axon regeneration [17], esthetic reasons [18], and multiple dental therapies [19, 20]. However, whether LLLT can promote primary gingival healing under the influence of BPs and prevent MRONJ needs to be further explored.

The aim of the study was to evaluate the effects of LLLT on promoting gingival wound healing in extraction sockets of MRONJ-like mice and preventing the occurrence of MRONJ. Furthermore, the underlying mechanisms were explored.

## Material and methods

### Patients

Five MRONJ patients, diagnosed according to AAMOS criteria [1], and five healthy controls were included in this study. MRONJ gingival samples and healthy gingival samples were obtained during the MRONJ-related surgeries or orthopedic surgeries at Peking University School and the Hospital of Stomatology. The collection process was standardized. Gingiva samples around the tooth were collected from patients in both groups. Specifically, the gingival samples of the MRONJ group were collected around the MRONJ-involved tooth, and the control gingival samples were collected around the healthy tooth during orthopedic surgeries. The harvested tissues were fixed in 4% paraformaldehyde and subjected to further analyses.

The present study was approved by the Institutional Review Board (IRB) of the Peking University Hospital of Stomatology (PKUSSIRB-202170184). All enrolled individuals provided written informed consent. The clinical features of healthy controls and MRONJ patients of the study population are shown in Additional file 1: Table S2.

### Antibodies and reagents

Zoledronate (Zol) was purchased from Sigma. An anti-OCN antibody was purchased from Abclonal. Anti-IL-1β, anti-TNF-α, and anti-IL-1RA antibodies were acquired from Abcam. Alexa Fluor 488 secondary antibody was purchased from Abcam. IHC-secondary antibodies were bought from ZSGB-BIO. IL-1 RA neutralizing and negative control antibodies were purchased from R&D Systems. IL-1β ELISA kit was purchased from Biolegend. Masson’s trichrome stain kit was obtained from Solarbio. Tartrate-resistant acid phosphatase (TRAP) stain kit was purchased from Sigma-Aldrich. Cell counting kit-8 was purchased from Dojindo.

### Animals

A 6–8-week-old male C57BL/6N mice were acquired from the Beijing Vital River Laboratory Animal Technology Co., Ltd. All the animals were housed in a specific pathogen-free facility with a relative humidity of 50 ± 1%, a temperature of 22 ± 1 °C, and a light/dark cycle of 12/12 h and were given standard chow and water. All animal experiments were approved by the Ethics Committee of the Peking University Health Science Center (LA2018265) and carried out in compliance with ARRIVE guidelines.

### Induction of an MRONJ-like animal model and treatment

In order to reflect the similar clinical disease state of MRONJ and further explore its mechanism, we built the MRONJ mouse model and compared the mouse gingival samples with human gingival tissues. Mice were randomly divided into the following groups (n = 5/group): (1) Ctrl group, which received a vehicle and no local treatment on the extraction site; (2) Zol group, which received a zoledronate and no local treatment on the extraction site; (3) Zol + LLLT group, which received a zoledronate and LLLT on the extraction site.

To build an MRONJ-like mouse model, mice were administered with zoledronate (500 μg /kg, SML0223, Sigma, United States) intraperitoneally twice a week for four weeks according to our preliminary animal experiments and previous studies [21]. After two weeks of drug treatment, tooth extraction of bilateral maxillary second molars was performed under general anesthesia using pentobarbital (50 mg/kg, P3761, Sigma, USA). The ctrl group or the Zol group received no local treatment for tooth sockets after tooth extraction. For local treatment, LLLT was locally employed to the tooth sockets immediately post extraction. In the subsequent rescue animal experiments, mice were randomly divided into four following groups (n = 5/group): (1) Ctrl group, which received a vehicle and negative control antibodies on the extraction site; (2) Zol group, which received a zoledronate and negative control antibodies on the extraction site; (3) Zol + LLLT group, which received a zoledronate, LLLT, and negative control antibodies on the extraction site; (4) Zol + LLLT + IL-1RA NAb group, which received a zoledronate, LLLT, and interleukin-1 receptor antagonist neutralizing antibody (IL-1RA NAb) on the extraction site. After tooth extraction and/or LLLT treatment, IL-1RA NAb (10 μg per mouse, AF-480-NA, R&D, USA) was topically injected into the extraction sockets using a micro sampler (91234910, Solarbio, China) in the Zol + LLLT + IL-1RA NAb group. Negative control antibodies (10 μg per mouse, AB-108-C, R&D, USA) were topically injected into the extraction sockets using a micro sampler for the Ctrl group, Zol group, and Zol + LLLT group. The local treatment was repeated 2 and 4 days after the tooth extraction. At two weeks post-extraction, all mice were euthanized by cervical dislocation. Untreated healthy mice were euthanized as natural healing control. The maxillae were collected and fixed in 4% paraformaldehyde, after which the fixed maxillae were subjected to further analyses.

### LLLT

We used a low-level laser (Velure S9 980 – Lasering) with the main wavelength of 980 nm to treat the tooth extraction. Three LLLT sessions were performed at the tooth extraction site at 0, 2, and 4 postoperative days. The irradiation parameters were as follows: 0.5 W power; spot size of 0.007829 cm^2^; continuous operation mode; power intensity of 63.87 W/ cm^2^. Irradiation was employed at a single point with the laser tip 5 mm above dental extraction sites for 45 s.

### Micro-CT analysis

The fixed maxillae were scanned using micro-computed tomography (microCT) (60 kV, 2 mA, J. Morita Corp., Kyoto, Japan) to assess hard tissue repair in the tooth extraction sockets. The area of interest was chosen, and bone mineral density (BMD) and bone volume/total volume (BV/TV) were quantified by three-dimensional (3D) bone morphometric software (SIEMENS, Munich, Germany). Data were analyzed using Image J.

### Histology

The fixed maxillae, including the extraction sockets, were decalcified in 10% EDTA, paraffin-embedded, and sectioned using a microtome (4 μm thick slices). Also, the fixed human gingival samples were sectioned using a microtome (4 μm thick slices). Hematoxylin and eosin (H&E) staining was then conducted for histological observations of the gingiva and tooth extraction sites (Solarbio, China). Masson’s trichrome staining was conducted to observe the collagen fibers following the instructions (Solarbio, China). TRAP staining was conducted to indicate osteoclasts following the instructions (Sigma-Aldrich, USA).

### Immunohistochemistry and immunofluorescence staining

Immunochemistry-paraffin (IHC-P) staining and immunofluorescent Staining (IF) were conducted to study LLLT-induced primary gingival wound healing employing the following primary antibodies: anti-OCN (A6205, Abclonal); anti-IL-1β (ab9722, Abcam); anti-TNF-α antibodies (ab1793, Abcam), anti-IL-1RA (ab124962, Abcam). For IHC-P staining, sections were deparaffinized, rehydrated, and inactivated endogenous peroxidase activity with 3% H_2_O_2_ in dark for 20 min. Then, sections were incubated in 0.01 M sodium citrate buffer solution at 98 °C for 20 min. After cooling to room temperature, sections were incubated with anti-OCN, or IL-1β or anti-TNF-α antibodies overnight at 4 °C. Then, sections were stained with secondary antibodies (PV-9001, ZSGB-BIO or PV-9002, ZSGB-BIO) and a DAB kit (ZLI-9018, ZSGB-BIO).

For IF staining, sections were handled as before and incubated with anti-IL-1RA antibodies overnight at 4 °C, after which they were stained with Alexa Fluor 488 secondary antibodies (1:200, Abcam) for 1 h and counterstained with DAPI (ZLI-9557, ZSGB-BIO). Images were acquired with an Olympus microscope. Images were analyzed using Image J.

### RNA isolation and quantitative real-time polymerase chain reaction (qRT-PCR)

A high-throughput tissue crusher was used to lyse gingival tissue into a paste. RNAs were extracted from mice gingival tissues with TriZol Reagent (15,596,026, Invitrogen, Thermo Fisher Scientific, USA) and reverse-transcribed (Takara, Japan) into complementary DNA (cDNA). The resultant cDNAs were used for the following experiments. qRT-PCR was applied to detect the gene expressions with the ABI Prism 7500. The relative expression level of targeted genes was quantified with β-actin (used as the internal control) and calculated with the 2^–ΔΔCT^ method. Additional file 1: Table S1 shows all of the primer sequences.

### Enzyme-linked immunosorbent assay (ELISA) for assessing IL-1β from gingival tissues

A high-throughput tissue crusher was used to lyse gingival tissue into a paste. Tissue supernatant of mice gingival samples were collected. IL-1β in gingival tissues was detected with ELISA kits (432604, Biolegend) under the manufacturer’s instructions. Then, a microplate reader measured the absorbance at 450 nm (Elx808; BioTek).

### Cell culture, viability, and migration assay

HaCaT cells, obtained from the central laboratory of Peking University Hospital of Stomatology, were cultured in DMEM (Dulbecco’s Modified Eagle’s Medium, Sigma-Aldrich, USA) supplemented with 1% penicillin − streptomycin (Gibco, USA) and 10% fetal bovine serum (Gibco, USA) in a humidified atmosphere at 37^◦^C in 5% CO_2_. The effect of Zol on HaCaT cells was examined using the cell counting kit-8 (CCK-8) assay (Dojindo, Japan). HaCaT cells were seeded in 96-well plates at a density of 2 × 10^3^ cells per well and incubated with 200 μL of the complete medium overnight to allow them to attach. Then, the medium was replaced with a fresh medium containing 1, 5, 10, 25, 50 μM Zol. After 24 h, 10 µL CCK-8 solution was added to each well and incubated for 2 h, and the absorbance of each well was measured at 450 nm absorbance. The effect of LLLT on the migration of HaCaT cells treated with Zol was assessed using the migration assay. In six-well plates, HaCaT cells were seeded and divided into the following groups: (1) Ctrl group: no drugs treatment; (2) Zol group: cells were incubated with 10 μM Zol for 24 h; (3) Zol + LLLT group: cells were incubated with 10 μM Zol for 24 h and then received LLLT (Velure S9 980 – Lasering, 980 nm, 0.5 W delivering energy doses at 0.5 J/cm^2^ for 10 s). After incubation with 10 μM Zol for 24 h, a sterile 10-μl pipet tip was used to scratch three separate wounds through the cells. The cells were gently rinsed in PBS to remove floating cells and incubated in the medium at 37 °C, 5% CO_2_/95% air environment. Images of the scratches were taken using an inverted microscope (Olympus, Lake Success, NY) at 0, 24, 48, and 72 h of incubation. The wound closure percentage was quantified using Image J.

### Statistical analysis

Statistical significance was evaluated by using Analysis of Variance (ANOVA) or the Student’s t-test with GraphPad Prism 8.0 (GraphPad Software, USA). The data were presented as mean ± standard deviation (SD). If the two-tailed *P* values were < 0.05 (*), < 0.01 (**), < 0.001 (***), and < 0.0001 (****), the difference was considered to be statistically significant.

## Results

### LLLT accelerates gingival wound closure and promotes socket bone regeneration in MRONJ-like mice

**Fig. 1:**
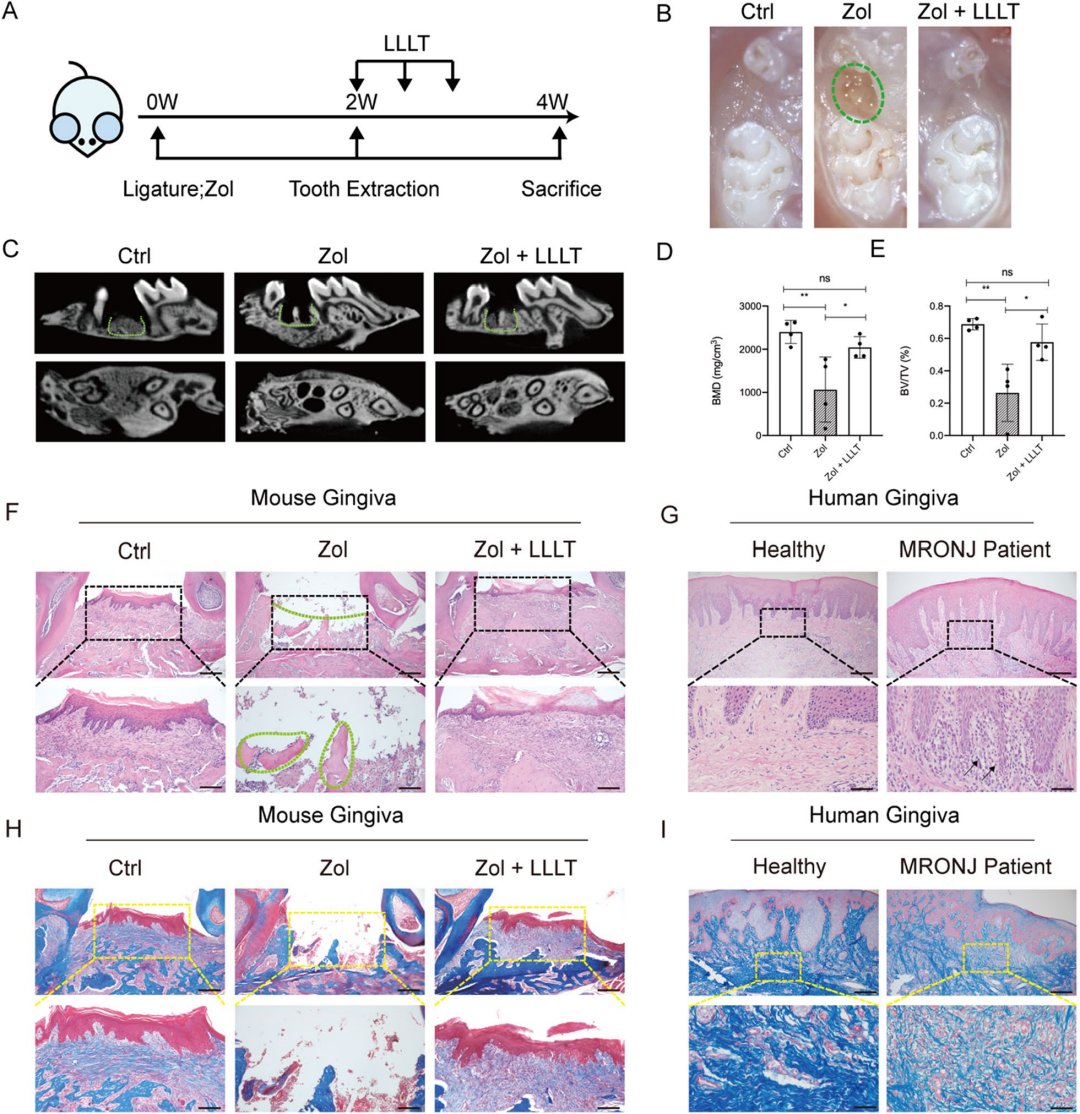
Effects of LLLT on MRONJ-like mouse model. **A** The study's experimental protocols are depicted in this diagram. **B** Representative intraoral photos. (Ctrl: non-drug treatment group; Zol: treated with Zol group; Zol + LLLT: treated with Zol and LLLT group). Green dotted lines represent unhealed gingiva. **C** MicroCT reconstructed 3D images of tooth sockets. Green dotted lines represent tooth sockets. **D**, **E** Quantification of BMD and BV/TV in each group. **F** H&E staining shows tooth extraction sockets-wound healing in each group. Black dotted boxes represent magnified boxed regions. Green dotted lines represent unhealed gingiva. Green dot lines represent necrotic bones. Scale bar = 200 μm (upper), scale bar = 100 μm (lower). **G** H&E staining shows the human gingiva of healthy and MRONJ patients. Black dotted boxes represent magnified boxed regions. Black arrows represent inflammatory cells. Scale bar = 200 μm (upper), scale bar = 50 μm (lower). **H** Masson’s trichrome staining shows tooth extraction sockets-wound healing in each group. Yellow dotted boxes represent magnified boxed regions. Scale bar = 200 μm (upper), scale bar = 100 μm (lower). **I** Masson’s trichrome staining shows collagen arrangement in human gingiva tissues. Yellow dotted boxes represent magnified boxed regions. Scale bar = 200 μm (upper), scale bar = 100 μm (lower). (*p < 0.05, **p < 0.01, ***p < 0.001, ****p < 0.0001)

Experiments were performed according to the schedule (Additional file 1: Fig. 1A). At 2 weeks post-extraction, stereoscope observations revealed that the tooth socket wounds were completely healed; consecutive mucosal covering was observed in both Ctrl and Zol + LLLT groups. At the same time, incomplete mucosal healing, lack of epithelial coverage, and exposed bone at the tooth socket wounds were seen in the Zol group (Additional file 1: Fig. 1B). MicroCT results further showed no obvious new bone regeneration at tooth-extraction sockets in the Zol group, while the Ctrl group and the Zol + LLLT group both showed ample newly formed bone (Additional file 1: Fig. 1C). The quantified data of microCT results indicated that BV/TV and BMD in alveolar sockets were significantly higher in the Ctrl group and Zol + LLLT group than in the Zol group (*P* < 0.05, Fig. 1D,E).

Histologically, the HE staining results showed intact epithelial coverage and adequate bone regeneration in sockets in the Ctrl and LLLT groups. In the Zol group, defective epithelial lining and necrotic bone with empty lacunae were observed (Fig. 1F and Additional file 1: Fig. S1A). In addition, Masson staining results showed more collagen deposition in the extraction sockets in the Zol + LLLT group compared to the Zol group (Fig. 1H). According to the results of TRAP staining, the Zol group had significantly fewer osteoclasts per bone marrow area (#/mm^2^), while LLLT application increased osteoclasts formation (Additional file 1: Fig. S1B and S1C). Additionally, immunohistochemical staining indicated significantly increased osteocalcin (OCN) expression in the Zol + LLLT group than in the Zol group (*P* < 0.05, Additional file 1: Fig. S1D and S1E).

Moreover, MRONJ patients had more inflammatory cell infiltration in the gingival tissue compared with healthy controls (Fig. 1G). Masson staining results showed that collagen arrangement was more disordered in MRONJ patients compared with healthy controls (Fig. 1I).

### LLLT suppresses pro-inflammation cytokines expression and enhances IL-1RA expression in gingival tissues at 2 weeks post-extraction

**Fig. 2:**
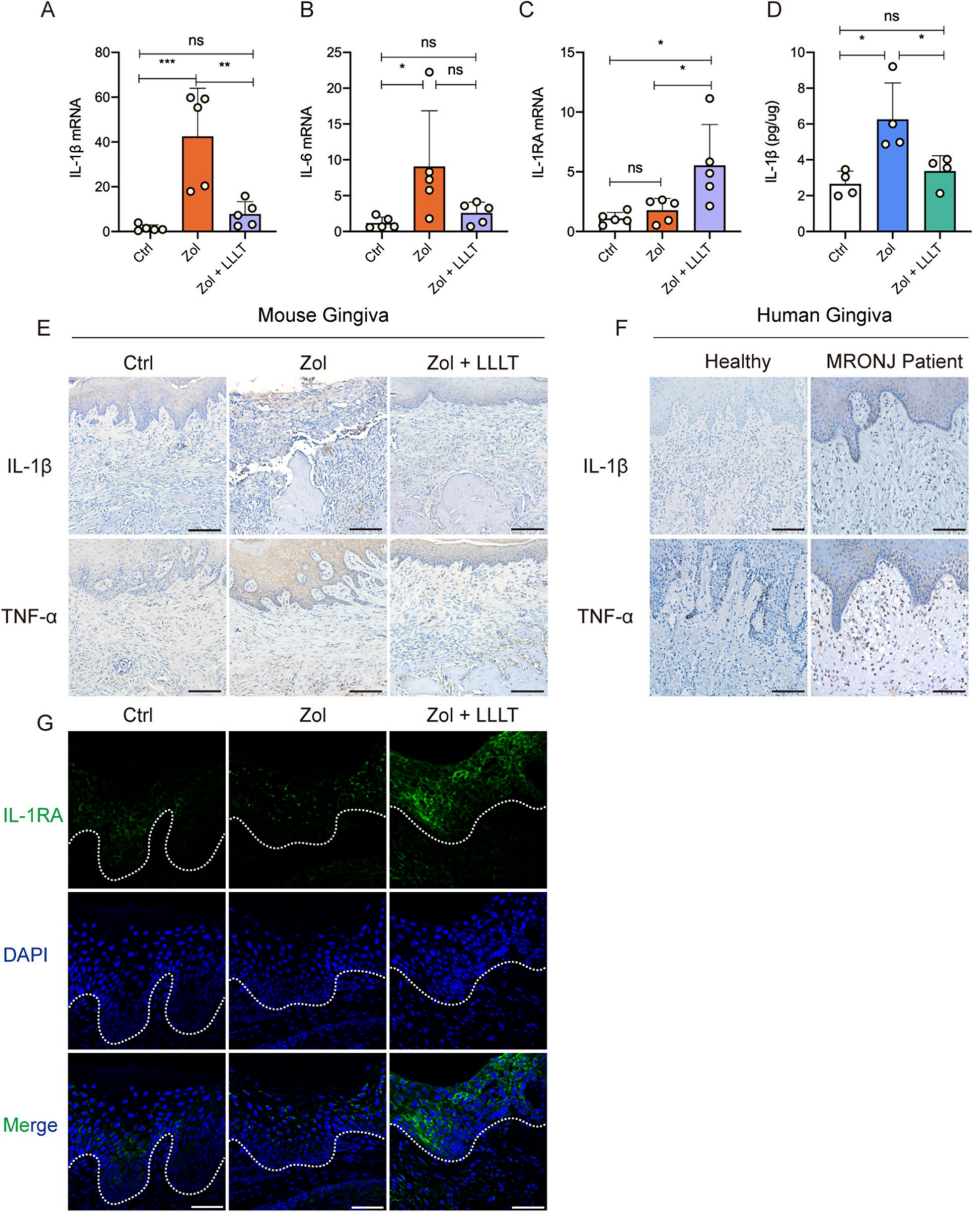
LLLT inhibited pro-inflammation cytokines, increased IL-1RA expression, and suppressed tissue inflammation in the gingiva. **A**–**C** Target gene mRNA expression levels in the gingival tissue of each group. **D** ELISA results of IL-1β levels in gingival tissue in each group. **E** Images of IL-1β and TNF-α IHC staining of the mouse gingival wound. Scale bar = 100 μm. **F** Images of IL-1β and TNF-α IHC staining of the human gingival wound. Scale bar = 100 μm. **G** Images of IL-1RA IF staining of the mouse gingival wound. White dotted lines represent the dividing line between the epithelium and the connective tissue. Scale bar = 50 μm. (*p < 0.05, **p < 0.01, ***p < 0.001, ****p < 0.0001)

To investigate the mechanism of how LLLT promoted gingival wound healing, mice gingiva was harvested at 11 days post-extraction. The qPCR results showed that IL-1β and IL-6 expression in the gingival tissues were downregulated, while IL-1RA expression was more upregulated in the Zol + LLLT group than in the Zol group (Fig. 2A–C). The results of ELISA further showed that LLLT decreased IL-1β expression in the gingival tissue compared with the Zol group (Fig. 2D). Immunohistochemistry indicated that IL-1β and TNF-α in the gingival tissue were upregulated in the Zol group compared with the Ctrl group. However, these factors were downregulated in the LLLT group (Fig. 2E). In addition, patients’ gingival samples of MRONJ lesions showed higher expression of IL-1β and TNF-α than the healthy controls (Fig. 2F). Immunofluorescence also indicated that IL-1 RA was highly expressed in the LLLT group compared with the Zol group (Fig. 2G).

### IL-1RA deficiency abolishes the effects of LLLT to accelerate gingival wound repair in MRONJ-like mice

**Fig. 3:**
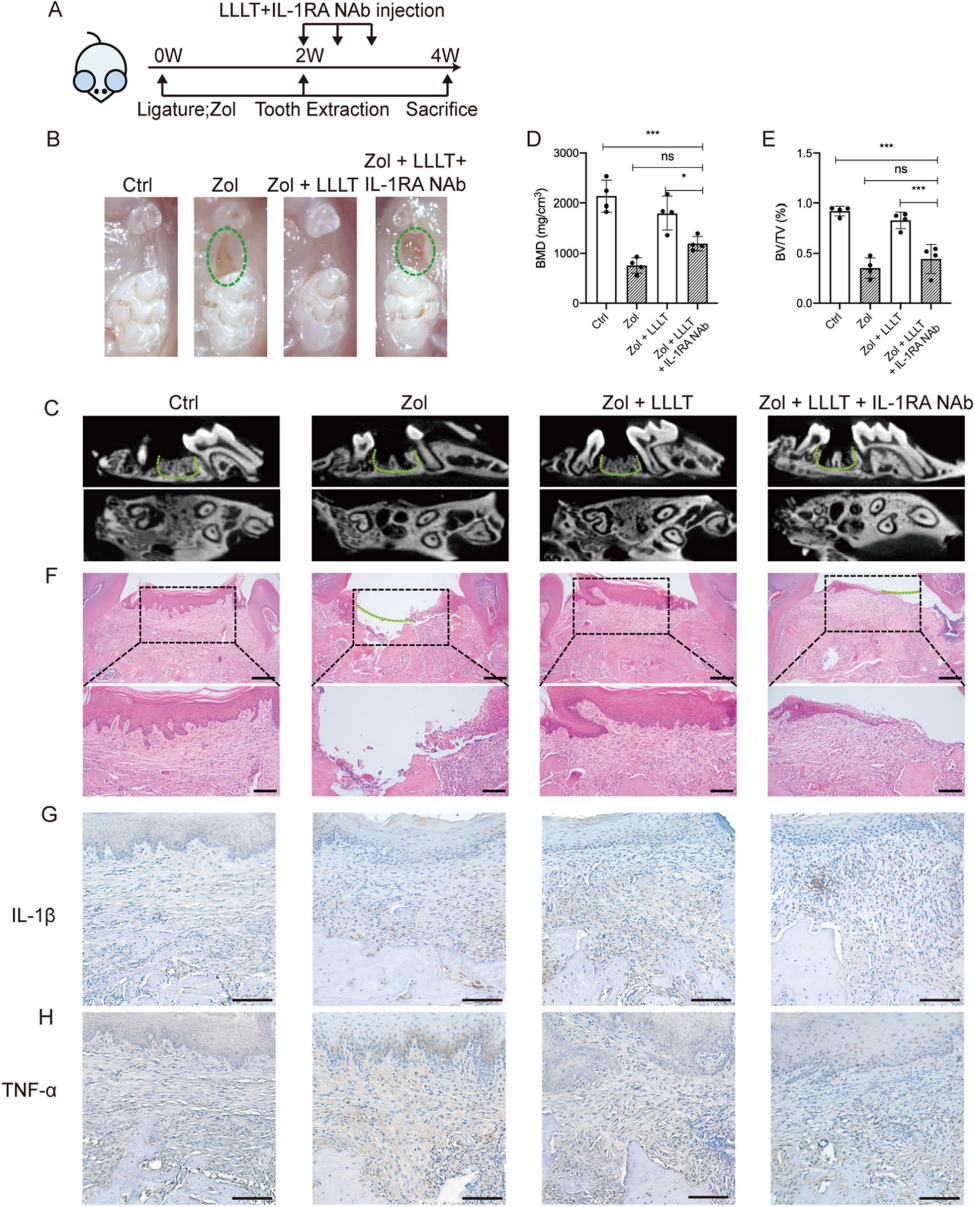
Application of IL-1RA NAb impairs the capacity of LLLT to promote gingival wound healing. **A** The study's experimental protocols are depicted in this diagram. **B** Representative intraoral photos. (Ctrl: non-drug treatment, but treated with negative control antibodies group; Zol: treated with Zol and negative control antibodies group; Zol + LLLT: treated with Zol, LLLT, and negative control antibodies group; Zol + LLLT + IL-1RA: treated with Zol, LLLT, and IL-1RA NAb group). Green dotted lines represent unhealed gingiva. **C** MicroCT reconstructed 3D images of tooth sockets. Green dotted lines represent tooth sockets. **D**, **E** Quantification of BMD and BV/TV in each group. **F** H&E staining shows tooth extraction sockets-wound healing in each group. Black dotted boxes represent magnified boxed regions. Green dotted lines represent unhealed gingiva. Scale bar = 200 μm (upper), scale bar = 100 μm (lower). **G**, **H** Images of IL-1β and TNF-α IHC staining of the mouse gingival wound. Scale bar = 100 μm. (*p < 0.05, **p < 0.01, ***p < 0.001, ****p < 0.0001)

A previous study has suggested that IL-1RA is important for wound healing [22]. Herein, we found increased IL-1RA expression in MRONJ lesions after LLLT. Then, we examined whether IL-1RA has a major part in LLLT promoting gingival wound healing. LLLT was performed on the tooth extraction sockets with or without IL-1RA NAb. The in vivo results showed that IL-1RA NAb abolishes the ability of LLLT to promote wound healing. Experiments were performed following the schedule (Fig. 3A). Stereoscope observations showed incomplete mucosal healing in the Zol + LLLT + IL-1RA NAb group compared with the Zol + LLLT group (Fig. 3B). MicroCT analysis further showed decreased bone formation, BMD, and BV/TV in the Zol + LLLT + IL-1RA NAb group compared with the Zol + LLLT group (P < 0.05, Fig. 3C–E). Histological analysis revealed that wound healing was delayed, and bone formation at the extraction site was reduced by using IL-1RA NAb (Fig. 3F and Additional file 1: Fig. S2A). The IL-1β and TNF-α expression increased again in the mouse gingiva in the Zol + LLLT + IL-1RA NAb group compared with the Zol + LLLT group, confirmed by immunohistochemistry (Fig. 3G, H). The above results suggest IL-1RA is involved in LLLT-induced primary gingival wound healing and MRONJ prevention.

### LLLT promotes Zol-treated HaCaT cells migration

**Fig. 4:**
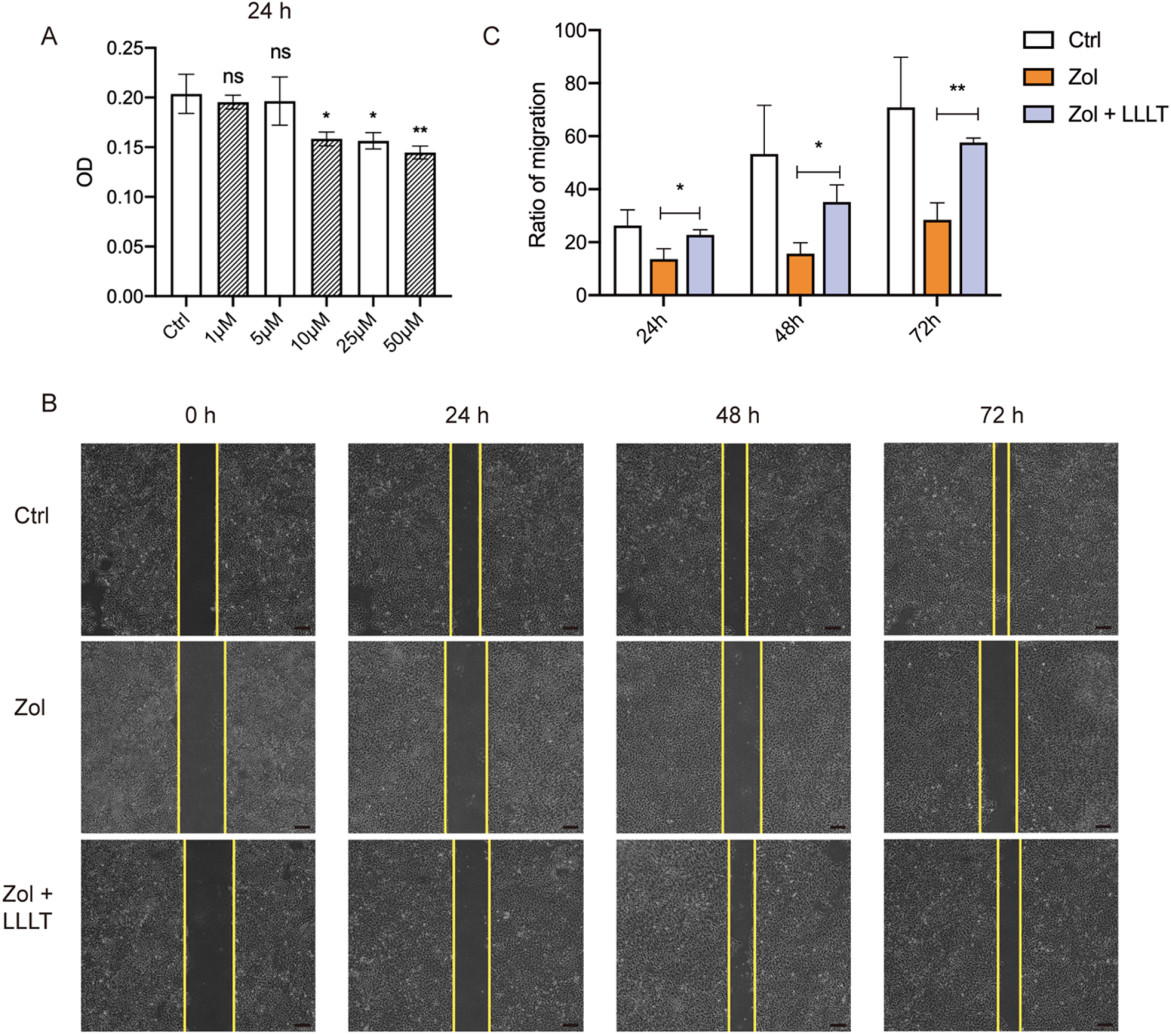
LLLT promotes Zol-treated HaCaT cell migration. (**A**) CCK8 showed the effects of Zol at different concentrations on HaCaT cell proliferation at 24 h. (**B**) The migration assay of HaCaT cells treated with Zol and LLLT. Scale bar = 200 μm. (**C**) Quantification of the migration rate at 24 h, 48 h, and 72 h. (*p < 0.05, **p < 0.01, ***p < 0.001, ****p < 0.0001)

A previous study has shown that BPs impede soft tissue wound healing by inhibiting epithelial cell proliferation and migration [23]. To detect the effects of Zol on HaCaT cells, we treated HaCaT cells with different concentrations of zoledronate, 1 μM, 5 μM, 10 μM, 25 μM, 50 μM, and found that 10 μM was the lowest concentration, at which HaCaT cells proliferation was inhibited at 24 h (P < 0.05, Fig. 4A). Thus, we chose 10 μM Zol for the migration assay. The migration assay demonstrated that Zol slows down the HaCaT cells’ migration rate, whereas LLLT significantly accelerates the migration rate at 24 h, 48 h, and 72 h (P < 0.05, Fig. 4B and 4C).

## Discussion

Since the first report on MRONJ was published in 2003 [24], different methods have been developed to prevent or treat MRONJ. Previous studies have suggested that promoting primary gingival healing or closure might be an effective method to prevent MRONJ [6, 8–10]. Methods to promote MRONJ soft tissue healing include platelet-rich fibrin injections, MSCs therapy, and so on [6, 25]. In this study, we found that LLLT might improve BPs-treated gingival wound healing via IL-1RA-mediated inflammation suppression. The primary wound closure provides a favorable environment for underlying bone regeneration of the tooth sockets and prevents the occurrence of MRONJ. Our results may help to clarify the mechanism of MRONJ and early clinical prevention of MRONJ by LLLT.

In MRONJ lesions, BPs-induced soft tissue toxicity impairs the functions of keratinocytes and fibroblasts and leads to delayed wound healing, which is a potential mechanism of MRONJ [9, 26]. Furthermore, periodontal ligament stem cells (PDLSCs) have remarkable regenerative potential and contribute to periodontal tissue regeneration [27]. Zoledronate might impair PDLSC’s viability, proliferation, and induce apoptosis, thus affecting gingival tissue healing [28, 29]. Oral mucosa acts as a defense against pathogenic bacteria or fungi for underlying bones. It also provides vital cytokines like bone morphogenetic proteins (BMPs), fibroblast growth factor (FGF), nuclear factor kappa-B ligand (RANKL), and TGF-β1 for bone metabolism [6, 9]. Loss of protection of intact mucosal coverage allows bacteria to invade the bone tissue, causing an inflammatory response, which, in turn, results in the development of osteonecrosis [30].

Primary gingival wound healing after tooth extraction can effectively reduce the onset of MRONJ [10]. Our previous studies suggested that TGF-β1-mediate early wound closure could promote the underlying bone repair process and prevent the occurrence of MRONJ [6, 31]. Moreover, other studies reported that LLLT could effectively promote wound healing, particularly in resistant wounds such as diabetic wounds [14], and inflammation-related wounds [32]. In diabetic wound healing, LLLT increases the serum anti-inflammatory cytokine IL-10 level and reduces the pro-inflammatory cytokines like IL-1β, and TNF-α levels in diabetic animals, restricting sustained inflammation and regulating the immune response [15]. Meanwhile, few studies suggested that LLLT promotes MRONJ wound healing [33–35]. In this study, we further found that LLLT promotes primary gingival wound healing and prevents MRONJ by reducing soft tissue inflammation, enhancing epithelial cell migration, and collagen formation.

**Fig. 5:**
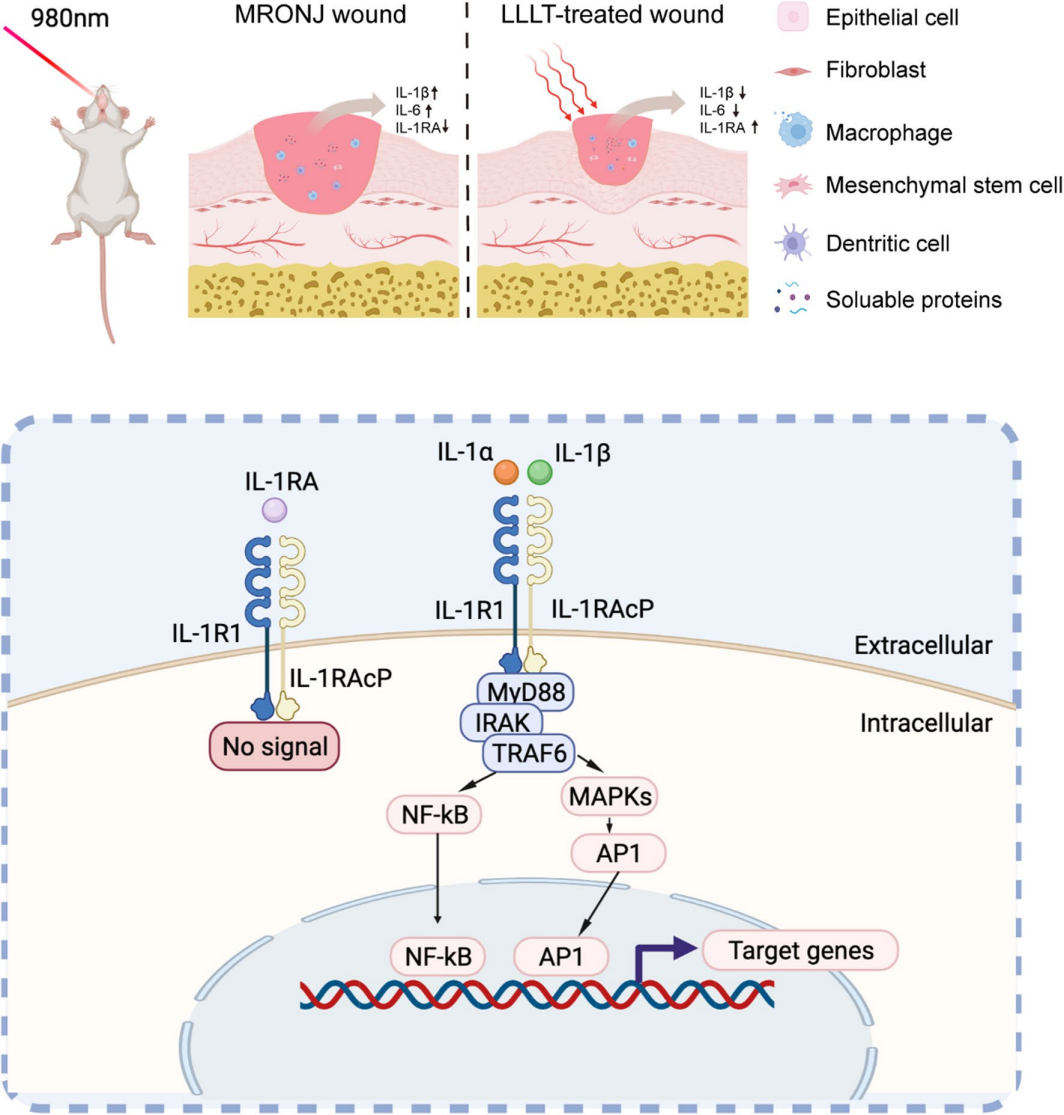
The schematic hypothesis of the function of LLLT to promote wound healing. Our results suggest that LLLT decreases IL-β and IL-6, and increases IL-1RA at the gingival wound, promoting wound healing and bone regeneration. The IL-1 signaling pathway is initiated when IL-1α or IL-1β binds to the members of the IL-1R1 family, which recruits MyD88, IRAK, and TRAF6 to activate NF-κB and MAPK and then controls the transcription of several inflammatory genes. IL-1RA can suppress this interaction competitively

IL-1RA might exert an important function in LLLT-meditated primary gingival wound healing. The inflammatory response has an important role during the wound-healing process. The excessive inflammatory response may disrupt normal tissue architecture and function and lead to impaired wound healing [36]. IL-1β is one of the typical inflammation factors and a crucial mediator of the inflammatory response, such as mitogen-activated protein kinase (MAPK) signaling and activating nuclear factor-kappa B (NF-κB) pathways, which controls the transcription of inflammatory genes, like IL-1, IL-6, TNF-α [37–39]. Many studies have reported that pro-inflammation cytokines like IL-1β, TNF-α, and IL-6 are over-expressed in the MRONJ lesions [40–42]. IL-1β overexpression prolongs the inflammatory stage and delays the soft wound healing, while IL-1RA inhibits the IL-1β-driven downstream signaling by combining the IL-1 receptor competitively and effectively blocking the IL-1β–driven inflammatory signals [43, 44]. Previous studies also suggested IL-1RA deficiency leads to delayed wound healing, while IL-1RA-upregulation promotes wound healing by inhibiting pro-inflammation effects [45, 46]. Our results indicated that LLLT might effectively suppress tissue inflammation by up-regulating IL-1RA expression, down-regulating IL-1β, TNF-α, and IL-6 in the gingival tissue, which increases collagen formation, promotes early gingival wound healing, and prevents MRONJ (Fig. 5).

Interestingly, we also found that LLLT improves bone regeneration and bone remodeling process. The histology results showed that LLLT might rescue the activated osteoclasts and increase the expression of OCN in the bone tissue. Moreover, the IL-1RA neutralizing antibody dramatically eliminated the effects of LLLT in promoting primary gingival wound healing. The rescue assay suggested that IL-1RA has a vital role in the LLLT-mediated prevention of MRONJ. Several studies found IL-1RA could enhance epithelial cell/ MSCs proliferation and migration capacities [47, 48]. Consistently, our in vitro studies confirmed that LLLT significantly accelerates Zol-treated HaCaT cell migration. However, the mechanisms of LLLT increasing IL-1RA expression are not clear. The increase of IL-1RA may be due to the direct stimulation of LLLT or the subsequent response caused by LLLT-mediated elevation of other cytokines, like TGF-β1 or IL-4 [49–55]. Studies have also suggested that LLLT can upregulate IL-1RA in the keratinocytes and fibroblast co-culture system [56], or increase salivary levels of IL-1RA in the periodontitis model [57].

There are still some limitations in the present study. There are many factors affecting the healing process of the MRONJ extraction sockets, like angiogenesis, bone remodeling, and immune regulation [58–60]. In our study, we focus more on how LLLT promoted primary gingival wound healing and prevented MRONJ; however, more research is required to fully understand the comprehensive effects of LLLT.

## Conclusions

LLLT can improve primary soft tissue healing by IL-1RA-mediated tissue inflammation inhibition and epithelial cell migration, thus promoting underlying osseous tissue repair and preventing the development of MRONJ. This study sheds new light on how LLLT helps to prevent MRONJ and offers new ideas and strategies for treating other disorders caused by an IL-1β-mediated excessive inflammatory response in the wound.

## Supplementary Information

**Additional file 1. Table S1:** Primers of targeted gene. **Table S2:** Clinical characteristics of healthy controls and MRONJ patients. **Figure S1:** LLLT might promote bone regeneration of tooth extraction. **Figure S2:** Application of IL-1RA NAb impairs the capacity of LLLT to promote gingival wound healing.

## Data Availability

The datasets used and/or analysed during the current study are available from the corresponding author on reasonable request.

## Abbreviations

MRONJ: Medication-related osteonecrosis of the jaw
BPs: Bisphosphonates
LLLT: Low-level light therapy
PBM: Photobiomodulation
IL-1RA: Interleukin-1 receptor antagonist
IL-1β: Interleukin-1β
TNF-α: Tumor necrosis factor-α
Zol: Zoledronate
microCT: Microcomputed tomography
BMD: Bone mineral density
BV/TV: Bone volume/total volume
HE: Hematoxylin and eosin
TRAP: Tartrate-resistant acid phosphatase
OCN: Osteocalcin
ELISA: Enzyme-linked immunosorbent assay
NAb: Neutralizing antibody
TGF-β1: Transforming growth factor-beta-1
BMPs: Bone morphogenetic proteins
FGF: Fibroblast growth factor
RANKL: Nuclear factor kappa-B ligand
MAPK: Mitogen-activated protein kinase
NF-κB: Nuclear factor-kappa B
PDLSCs: Periodontal ligament stem cells
