# Supplementary material for: Low-level laser therapy prevents medication-related osteonecrosis of the jaw-like lesions via IL-1RA-mediated primary gingival wound healing
Source: BMC Oral Health. 2023 Jan 10;23:14. doi: 10.1186/s12903-022-02678-1 (PMC9832759; doi:10.1186/s12903-022-02678-1)
Supplement: Supplementary file 1 — Additional file 1. Table S1: Primers of targeted gene. Table S2: Clinical characteristics of healthy controls and MRONJ patients. Figure S1: LLLT might promote bone regeneration of tooth extraction. Figure S2: Application of IL-1RA NAb impairs the capacity of LLLT to promote gingival wound healing. [file 12903_2022_2678_MOESM1_ESM.docx]

**Additional File Content**

**Supplemental tables and figures**

**Table S1丨** Primers of targeted gene

| mRNA | primer pairs (5’-3’) |
| --- | --- |
| Actin (m) | forward CATGTACGTTGCTATCCAGGC |
|  | reverse CTCCTTAATGTCACGCACGAT |
| IL-1RA (m) | forward TAGACATGGTGCCTATTGACCT |
|  | reverse TCGTGACTATAAGGGGCTCTTC |
| IL-1β (m) | forward GAAATGCCACCTTTTGACAGTG |
|  | reverse TGGATGCTCTCATCAGGACAG |
| IL-6 (m) | forward GGCGGATCGGATGTTGTGAT |
|  | reverse GGACCCCAGACAATCGGTTG |

**Table S2丨**Clinical characteristics of healthy controls and MRONJ patients

| **No.** | **Age** | **Gender** | **Disease** | **Bisphosphonate** | **Dose(mg)** | **Duration** | **Group** |
| --- | --- | --- | --- | --- | --- | --- | --- |
| **Patient 1** | 35 | Female | Maxillary protrusion,  Mandibular retrusion | No | 0 | 0 | Control |
| **Patient 2** | 19 | Female | Mandibular protrusion | No | 0 | 0 | Control |
| **Patient 3** | 18 | Female | Mandibular retrusion | No | 0 | 0 | Control |
| **Patient 4** | 28 | Female | Maxillary protrusion,  Mandibular protrusion | No | 0 | 0 | Control |
| **Patient 5** | 17 | Male | Maxillary retrusion,  Mandibular protrusion | No | 0 | 0 | Control |
| **Patient 6** | 62 | Male | Multiple myeloma | Zoledronic acid | 4(/month) | 24months | MRONJ |
| **Patient 7** | 50 | Female | breast cancer | Zoledronic acid | 4(/months) | 21months | MRONJ |
| **Patient 8** | 67 | Female | Multiple myeloma | Zoledronic acid | 4(/2month) | 26 months | MRONJ |
| **Patient 9** | 51 | Female | breast cancer | Zoledronic acid | 4(/months) | 35months | MRONJ |
| **Patient 10** | 54 | Male | Multiple myeloma | Zoledronic acid | 4(/month) | 12 months | MRONJ |

**Figure S1**


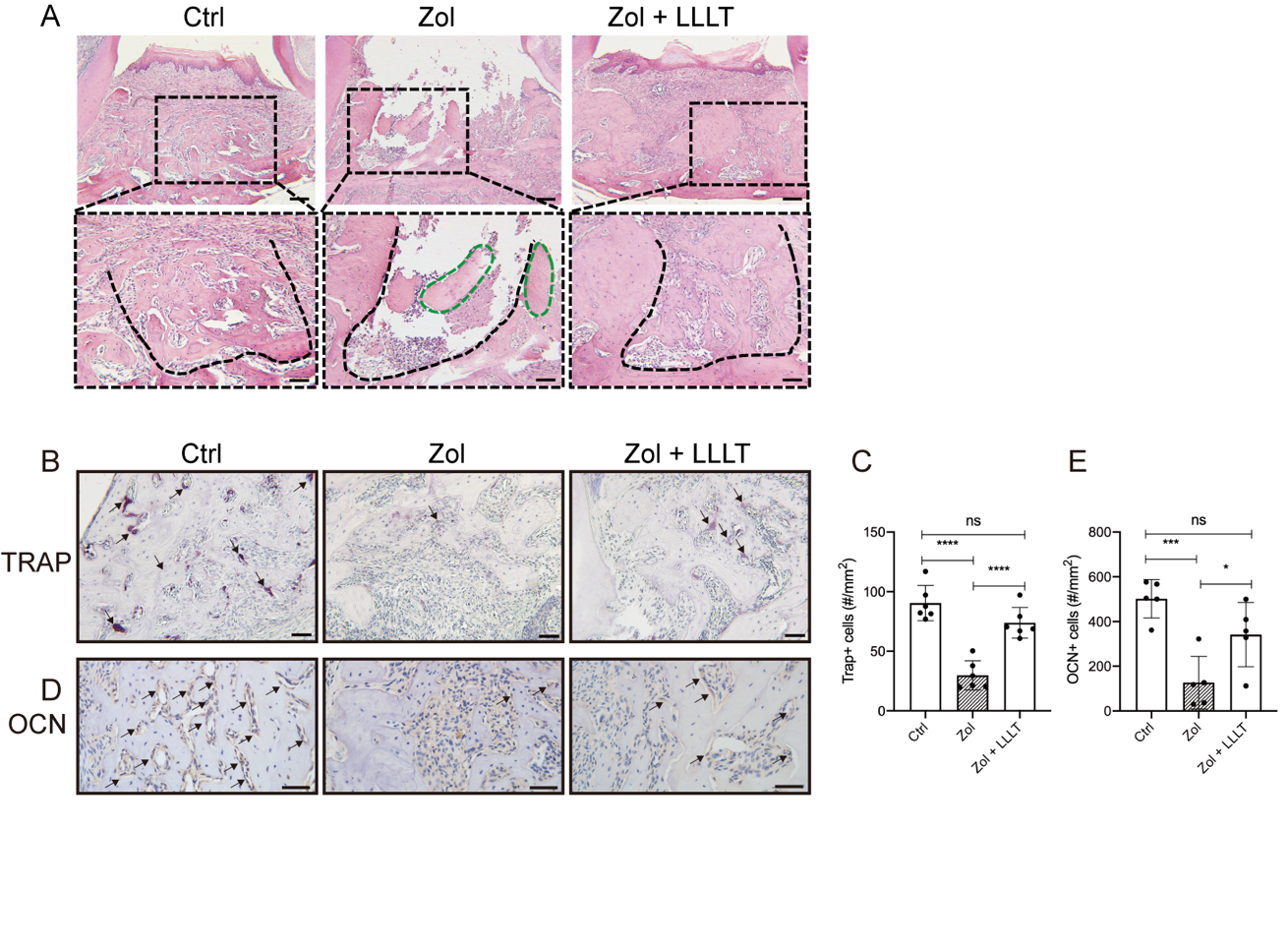
**Figure S1. LLLT might promote bone regeneration of tooth extraction. (A)** H&E staining shows tooth extraction sockets-wound healing in each group. Black dotted boxes represent magnified boxed regions. Black dotted lines represent tooth extraction sockets. Green dot lines represent necrotic bones. Scale bar=100μm (upper), scale bar=50μm (lower).  **(B)** TRAP staining indicates osteoclasts (black arrowheads) in tooth extraction sockets. Scale bar=50μm. **(C)** Quantification of osteoclasts cells in each group. **(D)** IHC staining of OCN shows OCN-positive cells (black arrowheads) in tooth extraction sockets. Scale bar=50μm. **(E)** Quantification of OCN-positive cells in each group. (∗p < 0.05, ∗∗p < 0.01, ∗∗∗p < 0.005, ∗∗∗∗p < 0.0001).

**Figure S2**

**
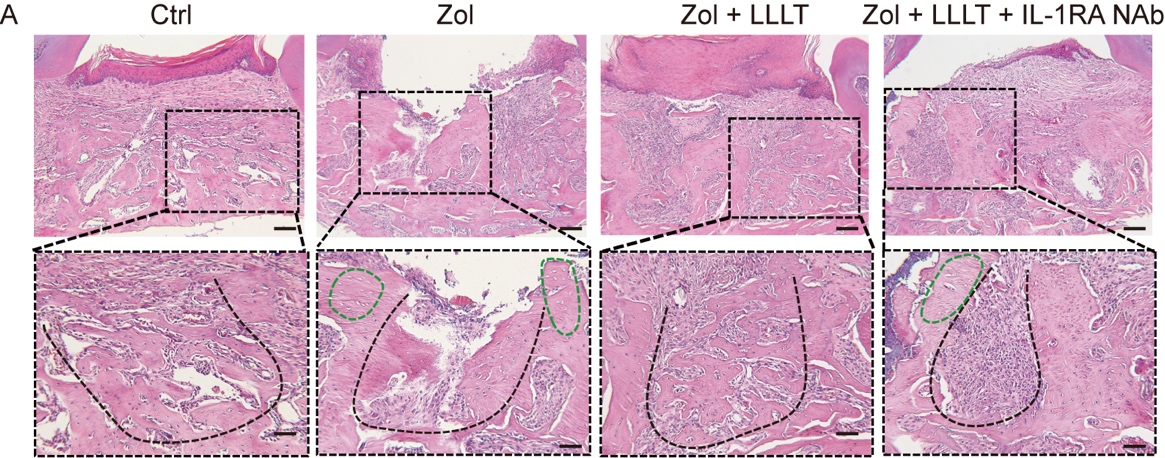
**

**Figure S2. Application of IL-1RA NAb impairs the capacity of LLLT to promote gingival wound healing*.*** H&E staining shows tooth extraction sockets-wound healing in each group. Black dotted boxes represent magnified boxed regions. Black dotted lines represent tooth extraction sockets. Green dot lines represent necrotic bones. Scale bar=100μm (upper), scale bar=50μm (lower).
